# Breaking the Saturated Vapor Layer with a Thin Porous Membrane

**DOI:** 10.3390/membranes12121231

**Published:** 2022-12-05

**Authors:** Yaoling Zhang, Fei Guo

**Affiliations:** School of Energy and Power Engineering, Dalian University of Technology, No. 2 Linggong Road, Dalian 116024, China

**Keywords:** membrane distillation, transmembrane flux capacity, saturated vapor layer, porous membrane, evaporation rate

## Abstract

The main idea of membrane distillation is to use a porous hydrophobic membrane as a barrier that isolates vapor from aqueous solutions. It is similar to the evaporation process from a free water surface but introduces solid–liquid interfaces and solid–vapor interfaces to a liquid–vapor interface. The transmembrane mass flux of a membrane-distillation process is affected by the membrane’s intrinsic properties and the temperature gradient across the membrane. It is interesting and important to know whether the evaporation process of membrane distillation is faster or slower than that of a free-surface evaporation under the same conditions and know the capacity of the transmembrane mass flux of a membrane-distillation process. In this work, a set of proof-of-principle experiments with various water surface/membrane interfacial conditions is performed. The effect and mechanism of membrane-induced evaporation are investigated. Moreover, a practical engineering model is proposed based on mathematical fitting and audacious simplification, which reflects the capacity of transmembrane flux.

## 1. Introduction

Evaporation is a basic interfacial thermodynamic process of converting water from the liquid phase to the vapor phase through the transfer of heat energy [1,2,3]. During the evaporation process, the escaping molecules accumulate as a vapor above the liquid, while many of the molecules return to the liquid. There is a saturated vapor layer above the liquid [4]. The saturated vapor pressure of water is a function of temperature, as described by the Clausius–Clapeyron equation [5,6]. The evaporation rate is mostly affected by the saturated layer or the saturated deficit among all the physical parameters. It can be significantly enhanced by breaking the saturated layer above the liquid.

Many of the applications for evaporation are intimately familiar to us. A typical application is a thermal-energy-based desalination. Saline water is heated up to a certain temperature to produce water vapor, which is then condensed to liquid water. Commonly employed thermal desalination processes include multi-stage flash (MSF) [7] distillation and multi-effect distillation (MED) [8,9]. The heated saline water is usually discharged to a chamber maintained below the saturation vapor pressure of the water. Breaking the saturated layer of water vapor is a significant and effective route to improve the specific distillation capacity. The specific evaporation rate of MSF distillation is about 300 kg/m^2^/h [10].

An interesting way of breaking the saturated layer above water is membrane distillation (MD). It is a thermally driven separation process in which separation is driven by phase change [11]. A hydrophobic membrane presents a barrier for the liquid phase, allowing the vapor phase to pass through the membrane’s pores [12,13]. The driving force of the process is the partial vapor pressure difference commonly triggered by a temperature difference [14,15]. The porous membrane directly contacting the heated water covers a certain area of the free water surface, which should suppress the specific evaporation rate. However, the thin membrane may also break or disturb the saturated vapor layer above the water surface [16], which may lead to an enhancement of evaporation.

It is important and necessary to ask these questions: (i) Does a porous membrane on the surface of water enhance or suppress the evaporation rate? (ii) Is there a theoretical maximum value of the transmembrane mass flux of an MD process? (iii) How is the capacity of the transmembrane mass flux of an MD process characterized? In this work, free-surface evaporation and transmembrane evaporation are compared under various conditions. A set of proof-of-principle experiments with various interfacial conditions is performed. Comparisons between membrane-induced evaporation and free-surface evaporation are investigated. The dominant factors, mechanisms, and mass transfer capacities are discussed in detail.

## 2. Experimental Section

### 2.1. Membranes

Polytetrafluoroethylene (PTFE) (Guangzhou Cleverflon New Material Technology Co., Ltd., Guangzhou, China) membranes were used for evaporation barrier and MD tests. The nominal mean pore size of the membranes was 0.22 μm. The surface morphology was characterized by Scanning Electron Microscope (SEM) (FEI, QUANTA 450, Hillsboro, OR, USA). The thickness was 60 μm, measured by a digital micrometer (211-101F, Guiling Guanglu Measuring Instrument Co., Ltd., Guiling, China). The apparent water contact angle of the test membranes was 155 ± 3°, measured by a goniometer (YIKE-360A, Chengde Yike Experimental Instrument Co., Ltd., Chengde, China). The porosity was around 85%, according to the gravimetric method [17].

### 2.2. Evaporation Test System

**Figure 1:**
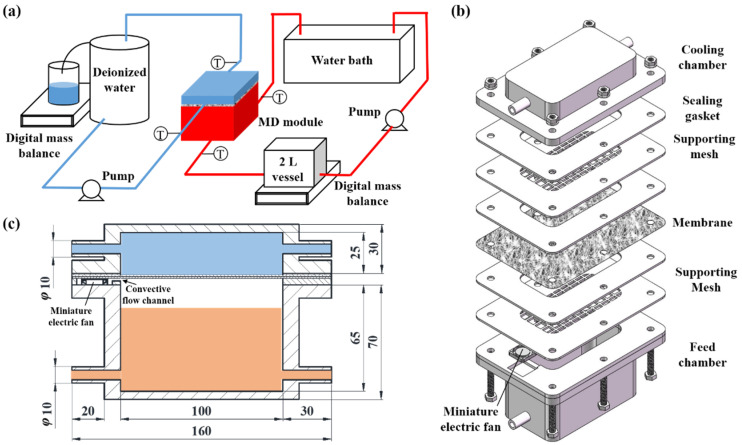
Schematic diagram and experimental module for the tests of evaporation rate with various conditions. (**a**) Schematic diagram of the experimental system that can be used for the tests of free evaporation, membrane-covered evaporation, and membrane distillation. (**b**) Exploded view of the experimental module. (**c**) Design and dimensions of the experimental test module.

The evaporation rates from water surface with and without a porous membrane coverage under various conditions were characterized by a test module made of polymethyl methacrylate (PMMA), as shown in Figure 1 and Figure S1 of Supplementary Materials. There are two chambers in the module, a heating chamber circulating at the lower side with hot solution as the feed, and a cooling chamber at the upper side circulating with cooling water. The sizes of the two chambers are 100 mm × 50 mm × 65 mm and 100 mm × 50 mm × 25 mm, respectively. The two chambers can be separated and assembled to test the evaporation rates with different configurations. In the sealing gasket, there is a small void space (4 mm × 4 mm × 2 mm) connected to the head space of the heating chamber. A miniature electric fan (Figure S1 of Supplementary Materials) was positioned in this space to generate a convection flow for disturbing the saturated layer above the liquid surface in the head space. To facilitate in-process visual observation, an aqueous solution of 2.0 wt% copper sulfate (CuSO_4_) (Tianjin Kemiou Chemical Reagent Co., Ltd., Tianjin, China) was circulated in the heating chamber, because of its blue color. Deionized water was circulated in the cooling chamber. The two circulations can be distinguished based on color. Peristaltic pumps (BT100-2J, Baoding Longer Peristaltic Pump Co., Ltd., Baoding, China) were used to circulate the liquids slowly and smoothly, with flow rates of 300 mL/min to avoid fluctuation at the interface. The reported values of this work were averaged over three times measurements.

### 2.3. Free-Surface Evaporation

**Figure 2:**
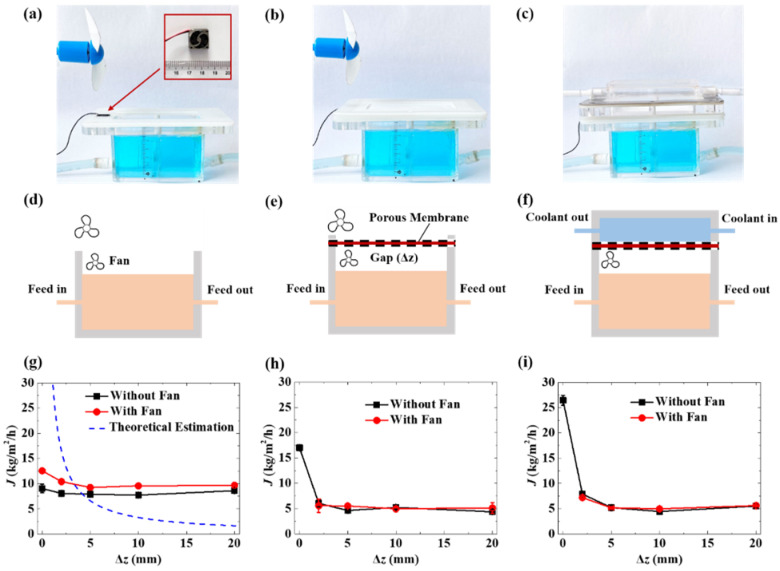
Digital photos, schematic diagrams, and corresponding relations between evaporation rate and gap distance. (**a**,**d**,**g**) Free-surface evaporation, (**b**,**e**,**h**) membrane-covered evaporation, (**c**,**f**,**i**) membrane distillation. The temperature of feed in the chamber was maintained at 80 ± 1 °C, by circulating the solution through the chamber and a heating water bath with a slow flow rate at 300 mL/min to avoid surface fluctuation. The temperatures of ambient air and coolant were maintained at 23 ± 1 °C. The inset figure is a miniature electric fan that generated convection flow to disturb the saturated layer above the liquid surface in the head space.

As shown in Figure 2a, the free-surface evaporation was tested using a module without a cooling chamber. The surface of the copper sulfate solution in the chamber was exposed to the atmosphere. The surface area for evaporation was 50 cm^2^. The temperature of the copper sulfate solution as the feed was maintained at 80 ± 1 °C by circulating it through the chamber and a heating water bath with a slow flow rate at 300 mL/min to avoid surface fluctuation. The temperature of ambient air was 23 ± 1 °C. The distance between the liquid surface and the upper edge of the chamber varied from near-zero to 20 mm, by tuning a valve on the circulating tube. To disturb or reduce the saturated layer above the water surface, a miniature electric fan (Figure S1 of Supplementary Materials) forced a convective flow onto the test surface, which reduces gas-phase transport resistance.

### 2.4. Membrane-Covered Evaporation

To investigate the effect of a porous membrane on evaporation, a membrane was supported by meshes and positioned above the pool surface along the top edge of the heating chamber, as shown in Figure 2b. The surface area of the porous membrane was 50 cm^2^. A miniature electric fan generated convection flow above the liquid surface and below the membrane, which maintained the premembrane concentration as approximately equal to the saturated water vapor. The temperatures of the feed solution and ambient air were maintained at 80 ± 1 °C and 23 ± 1 °C, respectively. There was also an electric fan above the membrane, generating convection flow to minimize gas-phase mass transport barriers and isolate the test membrane’s resistance. In the heating chamber, the gap between the porous membrane and the liquid surface varied from zero to 20 mm.

### 2.5. Membrane Distillation

In the MD tests, the two chambers were assembled together with the porous membrane as a barrier isolating the CuSO_4_ solution (feed) and deionized water (coolant), as shown in Figure 2c. The flow rates of the feed and coolant were 300 mL/min. The temperatures of feed and coolant were maintained at 80 ± 1 °C and 23 ± 1 °C, respectively. The temperatures at inlets and outlets of the chambers were measured and recorded by thermal couples and temperature monitors. The mass changes of the feed and coolant were measured and recorded by two digital mass balances.

## 3. Results and Discussion

### 3.1. Free-Surface Evaporation and Transmembrane Evaporation

The process of evaporation from a free water surface is a classical thermodynamics question and has attracted the attention of engineers and scientists for a long time. Many efforts have focused on the evaluation of the evaporation rate from a free water surface, including theoretical derivations, simulations, and experimental estimations. Among all the studies, the driving force of evaporation is recognized as the vapor pressure gradient near the water surface, which follows the same mass transfer principle to the MD processes.

(1) $$J=B\left(p_{1}-p_{2}\right)$$

where *J* is the evaporation rate or mass transfer flux, *p*_1_ and *p*_2_ are the values of the vapor pressure related to their local temperature governed by the Antoine equation, and *B* is the mass transfer coefficient. For a given condition with certain water and ambient temperatures, the *B* factor describes the capacity of mass transfer.

To study the effect of porous membranes on the evaporation process, a set of proof-of-principle experiments with various interfacial conditions is performed, as shown in Figure 2. Figure 2d illustrates a free-surface evaporation process that can be characterized by the one-dimensional steady-state diffusion process (Equation (2)) [16], which is also described in Figure S2 of Supplementary Materials. The evaporation rate is significantly affected by the gap (Δ*z*) between the water surface and the chamber’s upper edge. It is slightly affected by the temperature term, which is in the range of about 310 K to 350 K for common MD processes. The theoretical estimation (dash line) indicates the suppression of the potential evaporation rate by the saturated vapor layer above the water surface. By neglecting the saturated layer, Δ*z* = 0, the evaporation rate should be infinite.

(2) $$J=1.18\times 10^{-9}\frac{M_{w}}{R\Delta z}T^{0.75}\left(p_{1}-p_{2}\right)$$

where *J* is the evaporation rate or mass transfer flux from a free water surface. *p*_1_ and *p*_2_ are the saturated vapor pressure corresponding to the water surface and ambient temperatures, respectively. Δ*z* is the distance from the water surface to the chamber’s upper edge. *M_w_* is the molecular weight of water. *R* is the ideal gas constant. *T* is the temperature of the water in the chamber.

It is a classical thermodynamics phenomenon that a saturated vapor layer above the water surface suppresses the rate of evaporation and mass transfer near the liquid–vapor interface. The experimental data show that the evaporation rate is a finite value (~9 kg/m^2^/h) when Δ*z* = 0, which also reveals the existence of the saturated layer and its suppression effect. To reduce gas-phase transport resistance, a miniature electric fan forces a convective flow onto the test surface. The mass transfer flux increases slightly, indicating the disturbance and reduction, which still exist, of the saturated layer (Figure 2g). The values of the evaporation rate are on the same scale as the values in previous reports [18,19,20,21,22].

The vapor transport of the free water surface is based on molecular diffusion. It is clear that, for the free water surface, molecular diffusion is the only mechanism governing the vapor transportation, and it mainly depends on the water temperature. It governs the vapor transfer from any free surface of liquid. As a result, at any distance above the free water surface, the vapor transport is only governed by the molecular diffusion through the surface-adjacent saturated vapor layer (the mass transfer boundary layer).

As shown in Figure 2b,e,h, when a porous membrane is placed above the water surface, Δ*z* > 0, the mass transfer flux is smaller compared to the evaporation from the free water surface. This is because the porous membrane increases the total gas-phase transport resistance, by introducing the transmembrane diffusion resistance, and decreases the effective evaporation area of the water surface in the meantime. When the membrane directly contacts the water surface, the evaporate rate, which is the transmembrane flux under this condition, is larger than that without the membrane coverage. The porous membrane may break the original steady-state of the saturated layer, leading to the enhancement of the mass transfer flux from the liquid phase to the vapor phase.

To further investigate the effect of the porous membrane on evaporation, cooling water circulation is added into the experimental configuration (Figure 2c,f). The cooling water directly contacts the membrane on the downstream side. The intrinsically hydrophobic property makes the membrane non-wettable by liquid water. The vapor phase molecules diffuse through the membrane, condense into liquid immediately, and cannot come back to the feed side in the chamber. When there is a gap between the membrane and the hot water surface, the mass transfer flux is suppressed similarly to the case shown in Figure 2b,e,h. Notably, there is a significant increase in the mass transfer flux through the membrane, when the membrane contacts both the hot and cold sides, even with an unneglectable coverage of the evaporation surface. This is a typical direct contact membrane distillation (DCMD) process. It is believed that the membrane breaks or shortens the saturated layers on both the upstream and downstream sides and generates a new steady-state mass transfer process through the pores of the membrane. It is one of the promising routes for increasing the evaporation rate by MD compared to free-surface evaporation under the same operating conditions.

When the membrane covered the liquid surface, the saturated layer (boundary layer) is also covered by the membrane and remains just at the membrane pores. The transport mechanism of the vapor is governed by the Knudsen diffusion in addition to molecular diffusion (as given in Equation (3)). It is believed to provide an extra amount of vapor transport, as shown in Figure 2h,i.

DCMD, through the cooling water, offers a mechanism for withdrawing the vapor that has crossed the membrane faster than when just a membrane is placed above the water surface without cooling water circulation; thus, the flux of the DCMD was the highest, as shown in Figure 2h,i.

From another side, the presence of a miniature electric fan is an unaffected parameter when the membrane is covering the water surface; the distance change above the water surface (Δ*z*) is also an unaffected parameter, as shown in Figure 2h,i. While the miniature electric fan is an affective parameter of the free water surface because it acts to push the vapor away from the water surface, so the evaporation water is continuously compensating for the pushed vapor to keep the vapor pressure on the water surface, as shown in Figure 2g.

Moreover, with the existing distance above the water surface (Δ*z*), the mass transfer flux of the free water surface is larger than that of the two modes of the membrane-covered surface, and the latter had the same results for mass transfer flux because of the membrane barrier that the vapor escapes, even with the presence of the miniature electric fan, as shown in Figure 2g–i.

### 3.2. Diffusion Capacity through a Porous Membrane

When a porous membrane is infinitely close to or in direct contact with the surface of hot water, the saturated layer above the surface is disturbed or broken, and the dominant factor suppressing the evaporation rate and the transmembrane flux is the membrane. The transport behavior in the pores of a membrane is confined by the physical properties, including pore size, porosity, and thickness. According to the existing studies of MD, thermodynamic diffusion governs the capacity of transmembrane permeation flux. The mechanisms of both the Knudsen diffusion and molecular diffusion are expected to be operative in a common MD process, which can be considered as a transition diffusion (as shown in Figure S3 of Supplementary Materials).

(3) $$J={\left({\left(1.18\times 10^{-9}\frac{p}{p_{a}}\frac{\varepsilon }{\tau \delta }\frac{M_{w}}{R}T^{0.75}\right)}^{-1}+{\left(\frac{\varepsilon d}{3\tau \delta }{\left(\frac{8RT}{\pi M_{W}}\right)}^{\frac{1}{2}}\frac{M_{w}}{RT}\right)}^{-1}\right)}^{-1}\left(p_{1}-p_{2}\right)$$

where *ε* is porosity, *τ* is tortuosity, *δ* is thickness, and *d* is the mean pore size of the porous membrane. *p* and *p_a_* are the total pressure and the partial pressure of the air in the pores of the membrane, respectively. *D* is the diffusion coefficient of the vapor in the air, which can be calculated by the Fuller equation. *p*_1_ and *p*_2_ are the values of the saturated vapor pressure of the hot side and cold side, respectively.

In Equation (3), the tortuosity (*τ*) and thickness (*δ*) of a membrane relate to the diffusion path length, which acts as a resistance factor to diffusion. Assuming an infinitely thin porous membrane was applied in a DCMD process, the permeation flux could be a huge value. This is similar to the evaporation condition that directly from a free water surface without a saturated layer above it. Theoretically, it is a possible route to enhance the evaporation rate with a thin porous membrane by breaking or shortening the saturated layer.

**Figure 3:**
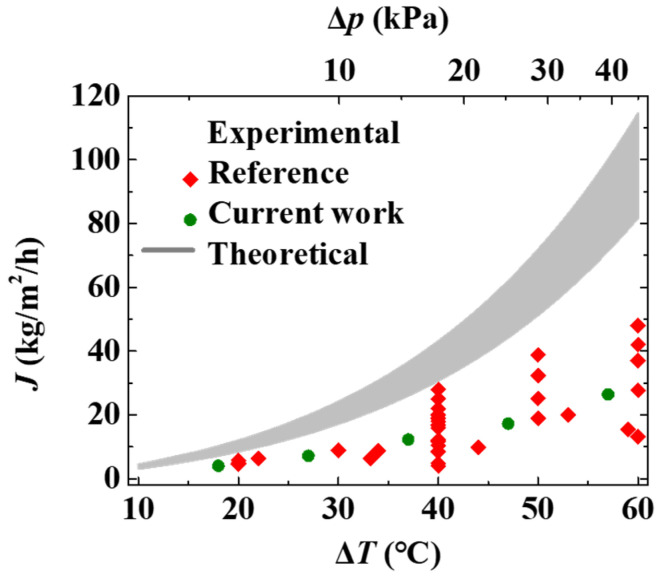
Theoretical estimation and experimental values of transmembrane mass flux of DCMD configuration. The shadow zone is the theoretical estimation of the flux values based on the transition diffusion model described by Equation (3). The values of thickness, porosity, and pore size are set as 50–70 μm, 0.7–0.8, and 0.2 μm, respectively.

However, the theoretical values of mass flux estimated based on the mechanism of thermodynamic diffusion are much larger than the values from the literature and experimental data of this work, as shown in Figure 3. The difference is more obvious at high temperatures. There should be resistance factors for evaporation and transport, other than the thermodynamic diffusion of the water molecules in the pores.

### 3.3. Dialectical Effect of Porous Membrane on Evaporation

**Figure 4:**
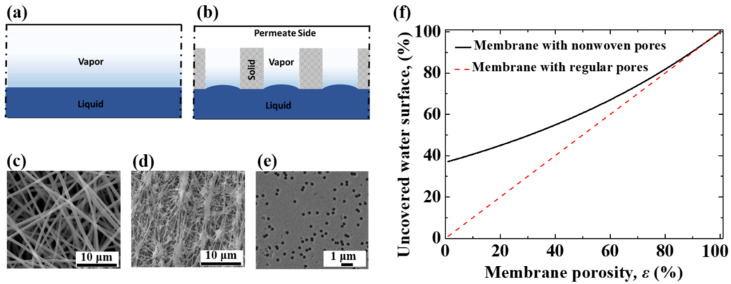
Interfaces introduced by a porous membrane. (**a**) Free-surface evaporation with the liquid–vapor interface. (**b**) Liquid-surface-contacting porous membrane introduces solid–liquid interface and solid–vapor interface. (**c**) Morphology of nonwoven fibrous membrane via electrospinning. (**d**) Morphology of commercial PTFE porous membrane via film-stretching. (**e**) Morphology of track-etched porous membrane with regularly cylindrical pores. (**f**) The ratio of the liquid surface uncovered by a porous membrane to the total liquid surface area is related to membrane porosity, according to Poisson theory.

In an MD process, the evaporation surface is usually taken as the surface directly contacting a membrane. However, the surface area of the membrane is not an effective area for evaporation when there is still a part of the water surface covered by the solid part of the porous membrane, which leads to a reduction in the apparent evaporation rate. For a free-surface evaporation process, there is only one interface, which is the liquid–vapor interface (Figure 4a). For an MD process, a porous membrane is loaded onto the water surface; the interfaces include the solid–liquid interface, the solid–vapor interface, and the liquid–vapor interface, as illustrated in Figure 4b. The solid–liquid interface acts as a barrier for free-surface evaporation. The apparent evaporation rate is enhanced at the liquid–vapor interface but is suppressed at the solid–vapor interface and solid–liquid interface. The porous membranes commonly applied in MD processes are fibrous membranes with irregular pores, e.g., nonwoven fibrous membranes (Figure 4c,d). There are also investigations on membranes with regularly cylindrical pores (e.g., track-etched pores [23], as shown in Figure 4e) for MD application. Nonwoven fibrous membranes usually have a larger specific porosity compared to membranes with regularly cylindrical pores.

According to the Poisson distribution theory, the ratio (Ω) of the uncovered water surface area (effective water surface for evaporation) to the total water surface can be estimated by Equation (4) [24]. It does not depend on the depth of the membrane immersed in the water or the height of the water in the pores but only depends on the porosity (*ε*) of the membrane. The uncovered area increases with the porosity of a porous membrane. For membranes with the same porosity, a nonwoven fibrous structure leaves more water surface area uncovered for evaporation, especially with a smaller porosity (Figure 4f). A larger uncovered area leads to a larger liquid–vapor interfacial area, resulting in a higher apparent evaporation rate, while a larger solid–liquid interfacial area results in a more significant barrier effect on the evaporation rate.

(4) $$\Omega =\left\{\begin{array}{l} \exp\left(-\left(1-\varepsilon \right)\right),\;\left(\mathrm{irregular}\;\mathrm{pores}\right)\\ \varepsilon ,\;\left(\mathrm{regular}\;\mathrm{pores}\right) \end{array}\right.$$

### 3.4. Transmembrane Flux Capacity of MD

Among all these evaporation processes, including free-surface evaporation and membrane-based evaporation, the fundamental driving force is the saturated vapor pressure difference, which is generated by the temperature difference between the bulk liquid water and the downstream target location. The *B* factor describes the capacity of the evaporation flux at various temperatures under a certain operating condition.

**Figure 5:**
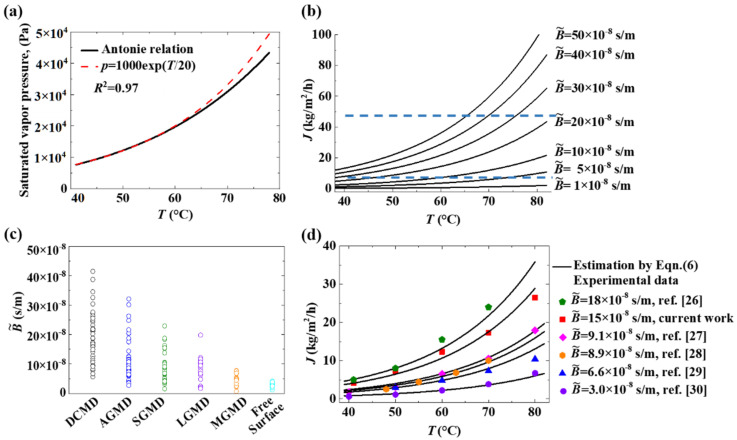
A practical coefficient characterizes the transmembrane flux capacity of MD. (**a**) The saturated vapor pressure estimated according to Antoine relation and its fitting lines as functions of local temperatures only, with the unit of °C. (**b**) The relation between transmembrane mass flux and feed temperature with various practical capacity coefficients ($\tilde{B}$), according to Equation (6). (**c**) The distribution of calculated $\tilde{B}$ values in various MD configurations and free-surface evaporation from the literature data (see Table S1 of Supplementary Materials). (**d**) The examinations of Equation (6) for the various literature data and the corresponding $\tilde{B}$ values.

The saturated vapor pressure can be estimated by the Antoine equation as a function of temperature only. The common MD processes are operated with the feed temperature in the range of 40–80 °C. The relation between the saturated vapor pressure and temperature can be well-fitted by Equation (5), which is a fitting trendline for the Antoine equation with an R-squared value larger than 0.95 (Figure 5a). It is also interesting to notice that the fitting equations are in the same form by using Celsius Temperature and Absolute Temperature, but only with different constants: 1000 and 0.001, respectively (Figure S4 of Supplementary Materials).

(5) $$p=1000\;\exp\;\left(T/20\right),\;\left(\mathrm{Temperature}:\;{}^{\circ}C\right)$$

The saturated vapor pressure is a small value in a relatively low temperature range (e.g., 20–30 °C, 3–5 kPa) but a significantly larger value in a relatively high temperature range (e.g., 60–90 °C, 20–90 kPa). Commonly, in an MD process, the temperature at the cooling side is in the range of 20–30 °C. It should be practicable to neglect the term of the saturated vapor pressure in a low temperature range. The flux change can be revealed by a corrected $\tilde{B}$ value in Equation (6), for further simplification.

(6) $$J=3.6\times 10^{6}\tilde{B}\exp\;\left(T/20\right),\;\left(\mathrm{Temperature}:\;{}^{\circ}C\right)$$

where the unit for *J*, the transmembrane flux, is kg/m^2^/h, the unit for the pressure term is Pa, and the unit for $\tilde{B}$ is kg/m^2^/h/Pa or s/m.

At this point, $\tilde{B}$ is the practical coefficient indicating the capacity of the mass flux escaping from liquid water as the feed or the capacity of the transmembrane mass flux. Importantly, it is a parameter independent of temperature. It comprehensively integrates many inherent transport behaviors of free-surface evaporation or an MD process. Moreover, it reflects a useful engineering property for the analysis and characterization of transmembrane mass flux.

As shown in Figure 5d, the $\tilde{B}$ values fit the experimental data in this work and the reported data in the literature well [25,26,27,28,29]. To the best of our knowledge, for various MD processes and configurations, the $\tilde{B}$ values are generally in the range of 5 × 10^−8^ to 20 × 10^−8^ s/m (Figure 5b). Among the basic configurations of MD, DCMD is the simplest and most commonly used configuration capable of producing a reasonably high flux. The reported values of mass flux across the porous membrane vary in a wide range. Figure 5c shows the distribution of calculated $\tilde{B}$ values in various MD configurations from the literature data, which is described in detail in Table S1 of Supplementary Materials.

## 4. Conclusions

The saturated layer above the water surface is the dominant factor affecting the evaporation flux. To enhance the flux, introducing a porous membrane that directly contacts the water surface a reasonable route to breaking or shortening the saturated layer. In a DCMD configuration, a porous membrane introduces new liquid–vapor interfaces with a shortened saturated layer in the pores, which should enhance the evaporation flux. However, it also introduces new solid–liquid interfaces and solid–vapor interfaces, which suppress the mass flux due to the water surface coverage, vapor diffusion resistance, and the existence of the saturated layer in the pores. Therefore, the transmembrane mass flux in an MD process is still a finite value, but it shows significant potential with the development of thin porous material fabrication. it is a potential route to further enhance the transmembrane flux for a fixed water surface by applying much thinner porous membranes. Considering the many interacting parameters that affect the transmembrane flux in an MD process, a practical engineering equation was proposed based on mathematical fitting and audacious simplification, which was examined and deemed suitable to describe the values and trending of the MD processes. In addition, the temperature-independent $\tilde{B}$ factor reflects the capacity of the transmembrane flux in an MD process, which is a useful parameter for the analysis and estimation of MD performance in terms of mass flux.

## Data Availability

The data presented in this study are available on request from the corresponding author.

## Supplementary Materials

The following supporting information can be downloaded at: https://www.mdpi.com/article/10.3390/membranes12121231/s1, Figure S1: A lab-scale evaporation test system and the different assembly configurations of the core module for the tests of evaporation ratios; Figure S2: (a) Schematic diagram of evaporation from a free water surface as the interface between liquid and gas. (b) The mass transfer flux from a free water surface under various values of gap distance and RH values; Figure S3: The value of Knudsen number calculated from water vapor temperature and membrane pore size.; Figure S4: The saturated vapor pressure estimated according to Antoine relation and its fitting lines as functions of local temperatures only, with the unit of °C and K, respectively; Table S1: The values of transmembrane mass flux and the calculated $\tilde{B}$ values of various membrane distillation processes from related references. References [26,27,30,31,32,33,34,35,36,37,38,39,40,41,42,43,44,45,46,47,48,49,50,51,52,53,54,55,56,57,58,59,60,61,62,63,64,65,66] were cited in the Supplementary Materials.
