# Supplementary material for: Breaking the Saturated Vapor Layer with a Thin Porous Membrane
Source: Membranes (Basel). 2022 Dec 5;12(12):1231. doi: 10.3390/membranes12121231 (PMC9784513; doi:10.3390/membranes12121231)
Supplement: Supplementary file 1 [file membranes-12-01231-s001.zip › membranes-2064665-supplementary.pdf]

membranes

Supplementary Materials

Breaking the Saturated Vapor Layer with a Thin Porous Membrane

Yaoling Zhang and Fei Guo *

School of Energy and Power Engineering, Dalian University of Technology, No. 2 Linggong Road, Dalian 116024, China
* Correspondence: feiguo@dlut.edu.cn

Index for this document:

1. *Evaporation test system and the core module*

2. *Theoretical estimation of mass transfer flux for free surface evaporation*

3. *Mass transfer behavior across porous membrane*

4. *Theoretical estimation of water surface coverage by fibers*

5. *Fitting equations for transmembrane flux capacity of MD*

6. *Reference collection*

1. Evaporation Test System and the Core Module

The tests for evaporation rates of three configurations, including free surface evaporation, membrane covered evaporation, membrane distillation, were realized by a lab-scale experimental system, as shown in Figure S1a. The core module was made of polymethyl methacrylate (PMMA) which can be visualized in operation. The free surface evaporation (Figure S1b), membrane covered evaporation (Figure S1c), membrane distillation (Figure S1d) can be tested by the same module with different assembly configurations.

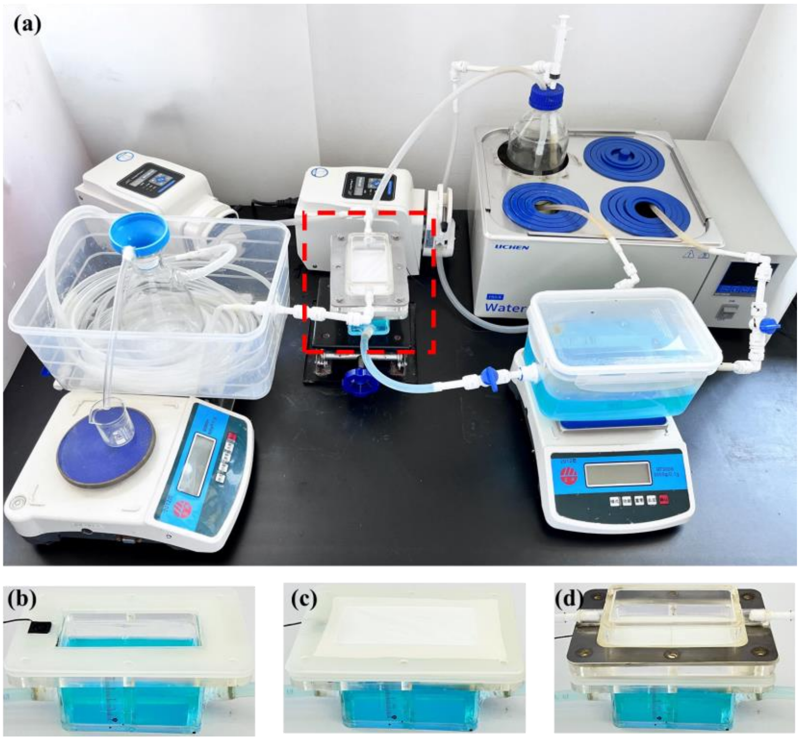

Figure S1. A lab-scale evaporation test system and the different assembly configurations of the core module for the tests of evaporation ratios. (**a**) The fully assembled system. (**b**) The assembly configuration for the test of free surface evaporation. (**c**) The assembly configuration for the test of membrane covered evaporation. (**d**) The assembly configuration for the test of membrane distillation. The inset figure is a miniature electric fan which generated convection flow to disturbing the saturated layer above the liquid surface in the head space.

2. Theoretical Estimation of Mass Transfer Flux for Free Surface Evaporation

As shown in Figure S2a, neglecting the effect of air motion on mass flux in evaporation from a free water surface. The evaporation rate from a free water surface can be evaluated by Equation (S1).

$$J_f = \frac{cDM_w}{\Delta z}\left(x_1 - x_2\right) \tag{S1}$$

where J_f is the evaporation rate or mass transfer flux from a free water surface. Δz is the distance between of water surface to the chamber upper edge. D is the diffusion coefficient of vapor and air. x is the molar fraction of vapor. c is the molar concentration of the mixed gas. The value of D can be calculated by Fuller formula (Equation (S2)).

$$D = 1\times10^{-7}\frac{T^{1.75}\left(\frac{1}{M_a}+\frac{1}{M_w}\right)^{\frac{1}{2}}}{p\left[\left(\Sigma v_a\right)^{\frac{1}{3}}+\left(\Sigma v_w\right)^{\frac{1}{3}}\right]^2} \tag{S2}$$

where p is the pressure and the unit of it is atm. $\sum v_a$ is the diffusion volume of air ($\sum v_a$ = 20.1). $\sum v_w$ is the diffusion volume of water vapor ($\sum v_w$ = 12.7). When p is 1 atm, the Equation S2 can be simplified as Equation (S3).

$$D = 1.18\times10^{-9}T^{1.75} \tag{S3}$$

The molar concentration of the mixed gas can be calculated by Equation (S4).

$$c = \frac{n}{V} = \frac{p}{RT} \tag{S4}$$

For the evaporation from a free water surface, p = 101325 Pa, R = 8.314 J/(mol·K). The unit of T is K. According to Dalton`s law, x can be calculated by Equation (S5).

$$x = \frac{c_{vapor}}{c} = \frac{\varphi p_s}{p} \tag{S5}$$

where p_s is the saturated vapor pressure of water. φ is the relative humidity. Then the Equation (S1) can be simplified as Equation (S6).

$$J_f = 1.18\times10^{-9}\frac{M_w}{R\Delta z}T^{0.75}\left(p_1-\varphi p_2\right) \tag{S6}$$

As shown in Figure S2b, the evaporation rate is dominated by the gap changes very slightly with the increase of relative humidity (RH). Therefore, the Equation (S6) can be further simplified as Equation (S7).

$$J_f = 1.18\times10^{-9}\frac{M_w}{R\Delta z}T^{0.75}\left(p_1-p_2\right) \tag{S7}$$

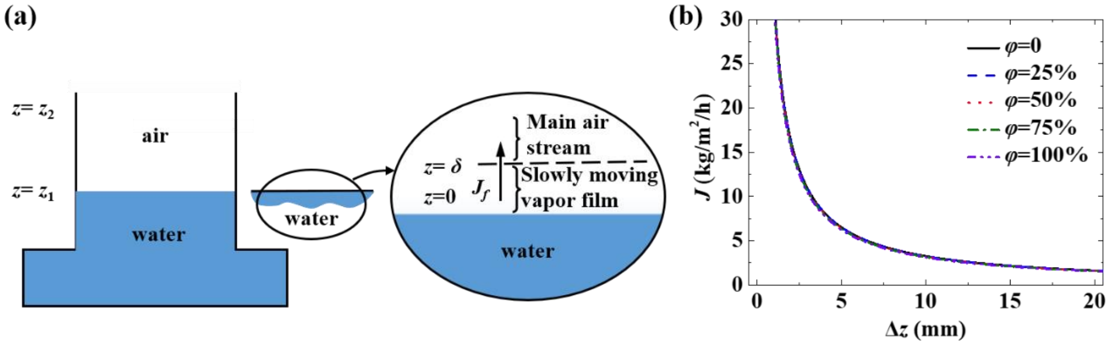

Figure S2. (**a**) Schematic diagram of evaporation from a free water surface as the interface between liquid and gas. (**b**) The mass transfer flux from a free water surface under various values of gap distance and RH values. The temperatures of liquid and ambient were set as 80 ˚C and 23˚C, respectively.

3. Mass transfer behavior across porous membrane

When the membrane pore size is 0.01–1 μm, the scale of *Kn* is 0.01–10 (see Figure S3). This indicates that the dominant mass transfer mechanism in the pores of MD process is transition diffusion.

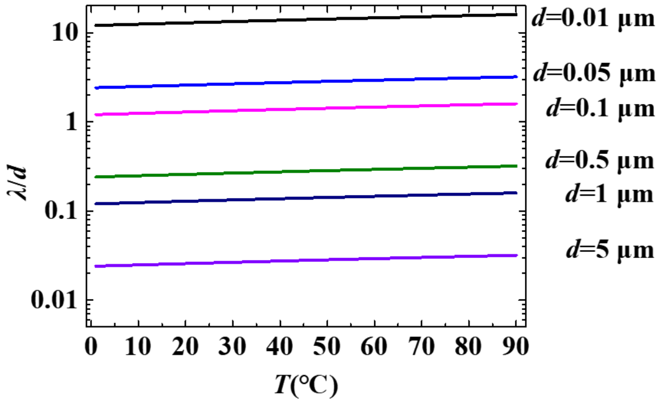

Figure S3. The value of Knudsen number calculated from water vapor temperature and membrane pore size.

4. Theoretical Estimation of Water Surface Coverage by Fibers

The shape of the fibrous membrane is square and the side length of it is l_m. The area of the fibrous membrane is $A_m = l_m^2$. When the thickness of the fibrous membrane is δ the volume of it is $V = A_m \times \delta$. The porosity of the fibrous membrane is ε. Then the volume of the fibers is $V_f = (1-\varepsilon) \times V$. Since the diameter of the fiber is in micron level, the bottom surface of the fiver is approximately a square with side length d. The bottom surface area of the fiber is $S = d^2$. The total layer of the fibers is $n = \delta/d$.

If the number of the fiber is one, the volume of it is V_f and the bottom surface area of it is S, then the length of this fiber should be $l = V_f/S = ((1-\varepsilon) \times V)/d^2$. Dividing the single fiber as the short length fibers. The length of the short length fiber is l_m. The number is $n_1 = l/l_m$. The number of the short fibers in each layer is $n_2 = n_1/n = ((1-\varepsilon) \times V)/(d \times l_m \times \delta)$.

If the fibers fall by an independent Poisson process that allows overlap. In this case, the effective area of the membrane with irregular pores is as follows:

$$\Omega = \exp(-\lambda A) = \exp\left(-\left(1-\varepsilon\right)\right) \tag{S8}$$

where λ is the density of short fibers in counts per square areal unit. A is the area of the short fibers ($A = l_m \times d$). ε is the porosity of the fibrous membrane.

5. Fitting Equations for Transmembrane Flux Capacity of MD

The relation between saturated vapor pressure and temperature can be well fitted by Equation (5) in the main text. The fitting trendlines of Antoine equation with the R-squared value larger than 0.95. The fitting equations present in the same form by using Celsius Temperature and Absolute Temperature, but only different constants as 1000 and 0.001, respectively.

$$p = \begin{cases} 1000\ \exp\left(T/20\right), & \left(\text{Temperature: ℃}\right) \\ 0.001\exp\left(T/20\right), & \left(\text{Temperature: K}\right) \end{cases}$$

$$J = \begin{cases} 3.6\times10^6 B\exp\left(T/20\right), & \left(\text{Temperature: ℃}\right) \\ 3.6\ B\exp\left(T/20\right), & \left(\text{Temperature: K}\right) \end{cases}$$

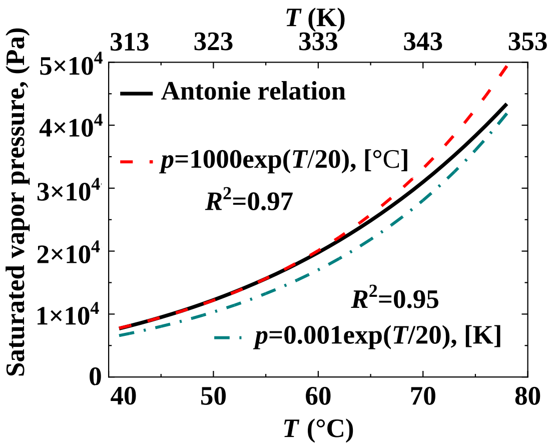

Figure S4. The saturated vapor pressure estimated according to Antoine relation and its fitting lines as functions of local temperatures only, with the unit of °C and K, respectively.

Table S1. The values of transmembrane mass flux and the calculated $\widetilde{B}$ values of various membrane distillation processes from related references.

	MD configuration	Reference
Figure 3		1–14
Figure 5c	DCMD	1–18
	AGMD	3, 19–26
	SGMD	26–32
	LGMD	23,33–38
	MGMD	33,39

References

1. Yao, M.; Woo, Y.C.; Tijing, L.D.; Shim, W.-G.; Choi, J.-S.; Kim, S.-H.; Shon, H.K. Effect of heat-press conditions on electrospun membranes for desalination by direct contact membrane distillation. *Desalination* **2016**, *378*, 80–91, https://doi.org/10.1016/j.desal.2015.09.025.
2. Tow, E.W.; Warsinger, D.M.; Trueworthy, A.M.; Swaminathan, J.; Thiel, G.P.; Zubair, S.M.; Myerson, A.S.; Lienhard V, J.H. Comparison of fouling propensity between reverse osmosis, forward osmosis, and membrane distillation. J. *Membr. Sci.* **2018**, *556*, 352–364, doi:10.1016/j.memsci.2018.03.065.
3. González, D.; Amigo, J.; Suárez, F. Membrane distillation: Perspectives for sustainable and improved desalination. *Renew. Sustain. Energy Rev.* **2017**, *80*, 238–259, https://doi.org/10.1016/j.rser.2017.05.078.
4. Singh, D.; Sirkar, K.K. Performance of PVDF flat membranes and hollow fibers in desalination by direct contact membrane distillation at high temperatures. *Sep. Purif. Technol.* **2017**, *187*, 264–273, https://doi.org/10.1016/j.seppur.2017.06.012.
5. Leitch, M.E.; Li, C.; Ikkala, O.; Mauter, M.S.; Lowry, G.V. Bacterial Nanocellulose Aerogel Membranes: Novel High-Porosity Materials for Membrane Distillation. *Environ. Sci. Technol. Lett.* **2016**, *3*, 85–91, https://doi.org/10.1021/acs.estlett.6b00030.
6. Wang, W.; Du, X.; Vahabi, H.; Zhao, S.; Yin, Y.; Kota, A.K.; Tong, T. Trade-off in membrane distillation with monolithic omniphobic membranes. *Nat. Commun.* **2019**, *10*, 1–9, https://doi.org/10.1038/s41467-019-11209-6.
7. Boo, C.; Lee, J.; Elimelech, M. Engineering Surface Energy and Nanostructure of Microporous Films for Expanded Membrane Distillation Applications. *Environ. Sci. Technol.* **2016**, *50*, 8112–8119, https://doi.org/10.1021/acs.est.6b02316.
8. Abdelrazeq, H.; Khraisheh, M.; Al Momani, F.; McLeskey, J.T.; Hassan, M.K.; Gad-El-Hak, M.; Tafreshi, H.V. Performance of electrospun polystyrene membranes in synthetic produced industrial water using direct-contact membrane distillation. *Desalination* **2020**, *493*, 114663, https://doi.org/10.1016/j.desal.2020.114663.
9. Khayet, M. Membranes and theoretical modeling of membrane distillation: A review. *Adv. Colloid Interface Sci.* **2011**, *164*, 56–88, https://doi.org/10.1016/j.cis.2010.09.005.
10. Deshpande, J.; Nithyanandam, K.; Pitchumani, R. Analysis and design of direct contact membrane distillation. *J. Membr. Sci.* **2017**, *523*, 301–316, https://doi.org/10.1016/j.memsci.2016.10.004.
11. Tijing, L.D.; Woo, Y.C.; Shim, W.-G.; He, T.; Choi, J.-S.; Kim, S.-H.; Shon, H.K. Superhydrophobic nanofiber membrane containing carbon nanotubes for high-performance direct contact membrane distillation. *J. Membr. Sci.* **2016**, *502*, 158–170, https://doi.org/10.1016/j.memsci.2015.12.014.
12. Kim, S.; Kim, S.; Ahmed, Z.; Cha, D.K.; Cho, J. Flux model for the membrane distillation process to treat wastewater: Effect of solids concentration. *J. Membr. Sci.* **2018**, *566*, 396–405, https://doi.org/10.1016/j.memsci.2018.09.018.

13. Anari, Z.; Sengupta, A.; Sardari, K.; Wickramasinghe, S.R. Surface modification of PVDF membranes for treating produced waters by direct contact membrane distillation. *Sep. Purif. Technol.* **2019**, *224*, 388–396, https://doi.org/10.1016/j.seppur.2019.05.032.
14. Ke, H.; Feldman, E.; Guzman, P.; Cole, J.; Wei, Q.; Chu, B.; Alkhudhiri, A.; Alrasheed, R.; Hsiao, B.S. Electrospun polystyrene nanofibrous membranes for direct contact membrane distillation. *J. Membr. Sci.* **2016**, *515*, 86–97, https://doi.org/10.1016/j.memsci.2016.05.052.
15. Muster-Slawitsch, B.; Dow, N.; Desai, D.; Pinches, D.; Brunner, C.; Duke, M. Membrane distillation for concentration of protein-rich waste water from meat processing. *J. Water Process Eng.* **2021**, *44*, 102285 https://doi.org/10.1016/j.jwpe.2021.102285.
16. Olatunji, S.O.; Camacho, L.M. Heat and Mass Transport in Modeling Membrane Distillation Configurations: A Review. *Front. Energy Res.* **2018**, *6*, https://doi.org/10.3389/fenrg.2018.00130.
17. Camacho, L.M.; Pinion, T.A.; Olatunji, S.O. Behavior of mixed-matrix graphene oxide – Polysulfone membranes in the process of direct contact membrane distillation. *Sep. Purif. Technol.* **2020**, *240*, 116645, https://doi.org/10.1016/j.seppur.2020.116645.
18. Chen, Y.; Zheng, R.; Wang, J.; Liu, Y.; Wang, Y.; Li, X.-M.; He, T. Laminated PTFE membranes to enhance the performance in direct contact membrane distillation for high salinity solution. *Desalination* **2017**, *424*, 140–148, https://doi.org/10.1016/j.desal.2017.10.007.
19. Singh, D.; Sirkar, K.K. Desalination by air gap membrane distillation using a two hollow-fiber-set membrane module. *J. Membr. Sci.* **2012**, 421-422, 172–179, https://doi.org/10.1016/j.memsci.2012.07.007.
20. Warsinger, D.E.; Swaminathan, J.; Maswadeh, L.A.; V, J.H.L. Superhydrophobic condenser surfaces for air gap membrane distillation. *J. Membr. Sci.* **2015**, *492*, 578–587, https://doi.org/10.1016/j.memsci.2015.05.067.
21. Ray, S.S.; Chen, S.-S.; Sangeetha, D.; Chang, H.-M.; Thanh, C.N.D.; Le, Q.H.; Ku, H.-M. Developments in forward osmosis and membrane distillation for desalination of waters. *Environ. Chem. Lett.* **2018**, *16*, 1247–1265, https://doi.org/10.1007/s10311-018-0750-7.
22. Warsinger, D.M.; Swaminathan, J.; Morales, L.L.; V, J.H.L. Comprehensive condensation flow regimes in air gap membrane distillation: Visualization and energy efficiency. *J. Membr. Sci.* **2018**, *555*, 517–528, https://doi.org/10.1016/j.memsci.2018.03.053.
23. Amaya-Vías, D.; López-Ramírez, J.A.; Gray, S.; Zhang, J.; Duke, M. Diffusion behavior of humic acid during desalination with air gap and water gap membrane distillation. *Water Res.* **2019**, *158*, 182–192, https://doi.org/10.1016/j.watres.2019.03.055.
24. Thomas, N.; Swaminathan, J.; Zaragoza, G.; Abu Al-Rub, R.K.; V, J.H.L.; Arafat, H.A. Comparative assessment of the effects of 3D printed feed spacers on process performance in MD systems. *Desalination* **2021**, *503*, https://doi.org/10.1016/j.desal.2021.114940.
25. Guo, F.; Servi, A.; Liu, A.; Gleason, K.K.; Rutledge, G.C. Desalination by Membrane Distillation using Electrospun Polyamide Fiber Membranes with Surface Fluorination by Chemical Vapor Deposition. *ACS Appl. Mater. Interfaces* **2015**, *7*, 8225–8232, https://doi.org/10.1021/acsami.5b01197.
26. M. Khayet, T. Matsuura, *Membrane distillation: Principles and applications*,1st ed. Elsevier, Amsterdam, Netherlands 2011.
27. Khayet, M.; Cojocaru, C.; Baroudi, A. Modeling and optimization of sweeping gas membrane distillation. *Desalination* **2012**, *287*, 159–166, https://doi.org/10.1016/j.desal.2011.04.070.
28. Khayet, M.; Cojocaru, C. Artificial neural network model for desalination by sweeping gas membrane distillation. *Desalination* **2013**, *308*, 102–110, https://doi.org/10.1016/j.desal.2012.06.023.
29. Wang, F.; Li, J.; Zhu, H.; Zhang, H.; Tang, H.; Chen, J.; Guo, Y. Physical modification of polytetrafluoroethylene flat membrane by a simple heat setting process and membrane wetting remission in SGMD for desalination. *Desalination* **2014**, *354*, 143–152, https://doi.org/10.1016/j.desal.2014.09.030.
30. Zhao, S.; Feron, P.H.; Xie, Z.; Zhang, J.; Hoang, M. Condensation studies in membrane evaporation and sweeping gas membrane distillation. *J. Membr. Sci.* **2014**, *462*, 9–16, https://doi.org/10.1016/j.memsci.2014.03.028.
31. Perfilov, V.; Fila, V.; Marcano, J.S. A general predictive model for sweeping gas membrane distillation. *Desalination* **2018**, *443*, 285–306, https://doi.org/10.1016/j.desal.2018.06.007.
32. Thakur, A.K.; Hsieh, I.-M.; Islam, M.R.; Lin, B.; Chen, C.-C.; Malmali, M. Performance of sweeping gas membrane distillation for treating produced water: Modeling and experiments. *Desalination* **2020**, *492*, 114597, doi:10.1016/j.desal.2020.114597.
33. Francis, L.; Ghaffour, N.; Alsaadi, A.A.; Amy, G.L. Material gap membrane distillation: A new design for water vapor flux enhancement. *J. Membr. Sci.* **2013**, *448*, 240–247, https://doi.org/10.1016/j.memsci.2013.08.013.
34. Essalhi, M.; Khayet, M. Application of a porous composite hydrophobic/hydrophilic membrane in desalination by air gap and liquid gap membrane distillation: A comparative study. *Sep. Purif. Technol.* **2014**, *133*, 176–186, https://doi.org/10.1016/j.seppur.2014.07.006.
35. Swaminathan, J.; Chung, H.W.; Warsinger, D.M.; AlMarzooqi, F.A.; Arafat, H.A.; Lienhard, J.H.V. Energy efficiency of permeate gap and novel conductive gap membrane distillation. *J. Membr. Sci.* **2016**, *502*, 171–178, doi:10.1016/j.memsci.2015.12.017.
36. Cheng, L.; Zhao, Y.; Li, P.; Li, W.; Wang, F. Comparative study of air gap and permeate gap membrane distillation using internal heat recovery hollow fiber membrane module. *Desalination* **2018**, *426*, 42–49, https://doi.org/10.1016/j.desal.2017.10.039.
37. Mahmoudi, F.; Date, A.; Akbarzadeh, A. Further investigation of simultaneous fresh water production and power generation concept by permeate gap membrane distillation system. *J. Membr. Sci.* **2018**, *572*, 230–245, https://doi.org/10.1016/j.memsci.2018.11.004.

38. Birgi, P.Y.; Ali, M.H.; Swaminathan, J.; Lienhard, J.; Arafat, H.A. Computational fluid dynamics modeling for performance assessment of permeate gap membrane distillation. *J. Membr. Sci.* **2018**, *568*, 55–66, https://doi.org/10.1016/j.memsci.2018.09.061.
39. Cai, J.; Yin, H.; Guo, F. Transport analysis of material gap membrane distillation desalination processes. *Desalination* **2020**, *481*, 114361, https://doi.org/10.1016/j.desal.2020.114361.
